# Impaired decision making following escalation of cocaine self‐administration predicts vulnerability to relapse in rats

**DOI:** 10.1111/adb.12738

**Published:** 2019-03-07

**Authors:** Paul John Cocker, Jean‐Yves Rotge, Marie‐Laure Daniel, Aude Belin‐Rauscent, David Belin

**Affiliations:** ^1^ Department of Psychology University of Cambridge Cambridge UK; ^2^ AP‐HP, Groupe Hospitalier Pitié‐Salpêtrière, Service de Psychiatrie d'Adultes Paris France; ^3^ Inserm U1127, CNRS UMR 7225, Sorbonne Université, Institut du Cerveau et de la Moelle, ICM Paris France

**Keywords:** addiction, cocaine, decision making, escalation, Iowa Gambling Task, relapse

## Abstract

Impairments in cost‐benefit decision making represent a cardinal feature of drug addiction. However, whether these alterations predate drug exposure, thereby contributing to facilitating loss of control over drug intake, or alternatively arise as a result of drug use and subsequently confer vulnerability to relapse has yet to be determined. Male Sprague‐Dawley rats were trained to self‐administer (SA) cocaine during 19 daily long‐access (12‐h) sessions; conditions reliably shown to promote escalation. One week after cocaine SA, rats underwent an extinction/relapse test immediately followed by conditioned stimuli–, stress‐, and drug‐primed reinstatement challenges. The influence of escalated cocaine intake on decision making was measured over time by four test sessions of a rodent analogue of the Iowa Gambling Task (rGT), once prior to cocaine exposure and then 1 day, 1 week, and 1 month after the last SA session. Substantial individual variability was observed in the influence of escalated cocaine SA on decision‐making performance. A subset of rats displayed pronounced deficits, while others showed unaffected or even improved performance on the rat Gambling Task (rGT) 24 hours after the last SA session. When challenged with a relapse test after 1 week of forced abstinence, animals that showed impaired decision making following SA displayed an increased propensity to respond for cocaine under extinction. These data suggest that decision‐making deficits in individuals with drug addiction are not antecedent to—but arise as a consequence of—drug exposure. Moreover, these data indicate that susceptibility to the deleterious effects of drugs on decision making confers vulnerability toward relapse.

## Graphical abstract

Whether alterations in cost‐benefit decision making that characterize drug addiction predate drug exposure and facilitate the loss of control over intake or instead result from drug use and subsequently confer vulnerability to relapse has yet to be determined. Here, we show that those rats whose decision‐making performance on a rat gambling task was impaired following a history of escalated cocaine self‐administration, but not prior to drug exposure, were more likely subsequently to relapse after a period of abstinence.

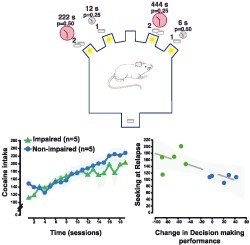

## 1 INTRODUCTION

Drug addiction encapsulates a constellation of behavioral alterations including impairments in executive functioning. Indeed, perturbations in cost‐benefit decision making have been canonically linked with multiple addictive disorders including addiction to cocaine,^1^ heroin,^2^ amphetamine,^3^ and alcohol,^4^ in addition to gambling disorder and polysubstance use.^5^, ^6^, ^7^, ^8^, ^9^ Impairments in decision making are associated with relapse following abstinence from drugs,^10^, ^11^ and the failure to acquire the optimal strategy during laboratory tests such as the Iowa Gambling Task (IGT) has been associated with dropout from treatment.^12^ The IGT is one of the most frequently used laboratory measures of “real‐world” decision making that has consistently highlighted impaired cost‐benefit decision making in individuals with addictive disorders.^13^, ^14^, ^15^, ^16^ However, despite reliable evidence linking impaired cost‐benefit decision making with substance and behavioral addictions, the causal relationship between decision‐making deficits and loss of control over drug use remains to be established. In other words, it is unclear whether deficits in decision making predate the onset of loss of control over drug use and consequently confer vulnerability toward loss of control over dug intake or whether the neurobiological sequela associated with this addictive process result in subsequent decision‐making deficits that then contribute to the individual vulnerability to relapse.

Animal models may be useful in addressing this question, in that they offer an opportunity, within longitudinal studies, to elucidate the relationship between decision making and drug exposure without the problematic issue of causality that is endemic to human studies. Multiple animal analogues of the IGT have been developed (see de Visser et al 17 for review); one of these paradigms has recently demonstrated that cocaine exposure exacerbates decision‐making deficits in animals characterized by their poor decision making on the task prior to drug exposure.^18^ Deficits in decision making were associated with an increased propensity to acquire responding for the drug‐paired cue acting as a conditioned reinforcer, but not to take more drugs.^18^ However, this study did not examine the relationship between decision making and the loss of control over drug intake, a hallmark feature of addiction^19^ or the propensity for animals to relapse. In contrast, George and colleagues employed a well‐established escalation of self‐administration (SA) procedure, which has been suggested to recapitulate several features of loss of control over intake,^20^, ^21^ to demonstrate that drug‐induced deficits in another executive function, namely, working memory, as measured by a delayed nonmatching to sample task, predict the rate of escalation of cocaine intake.^22^

Consequently, investigating whether differences in decision making at baseline confer vulnerability to escalation of subsequent intake or whether drug‐induced alterations in decision making contribute to propensity to relapse following abstinence would offer a meaningful insight into the contribution of decision‐making deficits to the development and maintenance of drug addiction.

Here, we utilized the rat Gambling Task (rGT), wherein like the human version, subjects choose between four “decks.” Two of the four available “decks” are risky, in that they offer larger initial gains but larger cumulative losses and are thus disadvantageous over the course of a session. In contrast, the other two “decks” are safe, in that they offer smaller immediate gains but smaller cumulative losses and are therefore advantageous. Animals are required to learn the contingencies and acquire the optimal strategy of learning to avoid the more tempting but ultimately disadvantageous options in order to maximize rewards and minimize punishments within a single time‐constrained session.^23^, ^24^ We assessed animals' baseline levels of decision making on a single rGT session prior to drug exposure. Rats were then tested again 1 day, 1 week, and 1 month after the cessation of long access to cocaine. Rats also underwent a relapse/extinction and reinstatement session following 1 week of forced abstinence. Consequently, we were able to examine whether individual differences in baseline decision making contributed to the increased acquisition or escalation of drug SA. Additionally, we looked at whether alterations in decision making following a history of escalated cocaine intake would be predicative of continued motivation to seek drug when it was no longer available and/or an increased propensity to relapse during a single extinction/reinstatement procedure.

## 2 METHODS AND MATERIALS

### 2.1 Subjects

Subjects were 16 adult male Sprague‐Dawley rats (Charles River, Arbresle, France) weighing approximately 250 at the start of the experiment. A week prior to behavioral training, rats were food restricted to 85% to 90% of their free feeding weight and maintained on approximately 20 g of rat chow per day. All animals were pair‐housed prior to surgery and single‐housed subsequently, in a climate‐controlled colony room maintained at 22 ± 1°C on a reverse light schedule (lights off 7 am). This research was regulated under the Animals (Scientific Procedures) Act 1986 Amendment Regulations (2012) following ethical review by the University of Cambridge Animal Welfare and Ethical Review Body (AWERB).

### 2.2 Rat version of the IGT

Testing took place as previously described^23^ in six standard five‐hole operant chambers enclosed within a larger wooden box equipped with exhaust fans that assured air renewal and masked background noise (Med Associates, Fairfax, Vermont). A five‐hole array was located along one wall, positioned 2 cm above a bar floor. Nose poke response into these apertures was detected via a horizontally positioned infrared beam located 1 cm from the entrance to each hole. Along the opposite wall, a food magazine was located 2 cm above the grid floor, and sugar pellets (Bio‐Serv, Flemington, New Jersey) were delivered via an external pellet dispenser. The boxes were controlled by a software written in Med‐PC on a computer running Windows 7. The habituation, training, and testing for the rGT were run in the same manner as previously described.^23^ In order to avoid neophobia, rats were first exposed to 20 sucrose pellets in their home cages before being habituated to the testing boxes during which rats received 60 pellets in the magazine. The next day, 60 pellets were delivered to the magazine on a 30‐second variable interval schedule. Rats were then trained to nose poke into one of the four lateral illuminated holes to receive a food pellet reward. Responses in the middle inoperative hole were recorded but had no programmed consequence. Sessions continued until rats obtained 100 pellets or 30 minutes elapsed. After two free‐choice training sessions, rats were given four forced‐choice 30‐minute sessions during which one of the four holes was active for 7 minutes 30 seconds on a pseudorandom schedule. Forced‐choice sessions were implemented to help animals avoid development of a side or hole bias. Subsequently, animals underwent two consecutive free‐choice sessions; the second of these was designed to expose the rats to higher incentive values. Thus, in the second of these, each nose poke in any of the four active holes resulted in the delivery of two pellets during the first half of the session and one pellet during the second half. During these last free‐choice sessions, any side preferences were recorded for each rat.

On the day of the rGT challenge, novel contingencies were introduced such that two of the holes were advantageous; they were associated with only one sugar pellet, but relatively short time‐out punishments of 6 or 12 seconds delivered with a probability of 0.5 and 0.25, respectively. The other two holes were disadvantageous; although they yielded a higher reward of two pellets, potential time‐outs were longer, lasting 222 or 444 seconds with respective probabilities of 0.5 and 0.25. The probability of receiving a time‐out punishment for each hole was fixed for the duration of the session. The test session lasted until rats obtained 250 pellets or 60 minutes had elapsed. A configuration was assigned to each rat: the side of the advantageous holes was counterbalanced with any side preference previously identified. Animals were initially tested prior to intravenous surgery and then 1 day, 1 week, and finally 1 month following the final extended access session. Prior to all subsequent rGT sessions, animals were rebaselined with two free‐choice sessions. During the first of these training sessions, a response in any of the four active holes delivered two pellets and one pellet on the second session. In subsequent rGT test sessions, advantageous holes were counterbalanced against any identified side preference from the two previous free‐choice sessions.

### 2.3 Intrajugular surgery

Rats were deeply anesthetized with intraperitoneal administration of ketamine (100 mg/kg; Ketalar, Panpharma, France) and xylazine (1 mg/kg; Rompun, Bayer, Puteaux, France), and all surgeries were conducted as previously described.^25^ A silastic catheter (internal diameter = 0.28 mm; external diameter = 0.61 mm; dead volume = 12 μL) was implanted in the right jugular vein. The catheter remained available through a nylon mesh sutured between scapulae. To prevent infection, rats received prophylactic antibiotics (10 mg/kg; Baytril, Bayer, Puteaux, France), 1 day prior to and 6 days post surgery. After surgery, rats were allowed to recover for 7 days. During this period, catheters were daily flushed with a saline solution containing unfractionated heparin (20 IU/mL).

### 2.4 Drugs

Cocaine hydrochloride (Cooper, Bordeaux, France) was dissolved in sterile 0.9% saline. The infusion dose of 250 μg/100 μL (approximately 0.8 mg/kg) was calculated as the salt.

### 2.5 Cocaine SA

All SA sessions took place as previously described,^20^ in standard chambers for operant conditioning (Med Associates), enclosed within a ventilated, sound‐attenuated box. Each chamber had two levers on the right wall located 5 cm from above the grid floor. A cue light was located above each lever, and the chamber could be illuminated via a central houselight. During SA, the indwelling catheters were attached to a metal spring‐covered swivel (Stoelting, Wood Dale, Illinois) connected to a Razel infusion pump (Semat Technical, Herts, UK). Levers were permanently designated as either active or inactive and counterbalanced between animals. Responses on the active lever delivered an infusion of cocaine (250 μg/100 μL/5.7 s) under a fixed ratio 1 (FR1) schedule of reinforcement, followed by a 20‐second time‐out period during which the houselight was switched off; both levers were retracted, and the cue light was illuminated above the active lever position. Responses on the inactive lever were recorded but had no programmed consequence. All rats initially acquired cocaine SA over daily 1‐hour sessions before subsequently being exposed to 12‐hour extended access sessions for 19 days, conditions previously shown to induce robust escalation of cocaine intake.^20^, ^21^

### 2.6 Relapse and reinstatement procedures

Seven days after the last SA session, rats were tested in the same boxes for a single 210‐minute extinction/relapse‐reinstatement session, similar to previously described.^20^, ^26^ The relapse test consisted of a 90‐minute extinction challenge during which both active and inactive levers were presented but pressing on either had no programmed consequences. This was followed by a 30‐minute conditioned stimuli (CS)–induced reinstatement test, at the onset of which the cocaine‐paired CS was presented noncontingently for 20 seconds. During the next 30‐minute period, cocaine‐paired CS presentations were contingent on active lever presses, under an FR1 schedule. The cue light above the active lever would illuminate for 2 seconds upon each active lever press, but no cocaine was delivered. At the end of this 30‐minute period, a noncontingent presentation of a 0.4‐mA footshock initiated another 30‐minute period over which nonreinforced responding was measured. Lastly, a noncontingent infusion of cocaine (250 μg/100 μL) was delivered at the start of the next 30‐minute reinstatement period in order to measure drug‐induced reinstatement.

### 2.7 Data and statistical analyses

During the rGT, as the utility within each pair of options was identical, choices of either advantageous option were pooled, as were choices from either disadvantageous option in order to generate a decision‐making score for each animal, as previously described.^23^, ^27^

Statistical analyses were performed with the StatSoft Statistica 9 package. Assumptions for normal distribution and homogeneity of variance were tested with the Kolmogorov‐Smirnov and Levene tests, respectively. Percent advantageous choice across rGT sessions was analyzed with a repeated‐measures analysis of variance (ANOVA) with session (four levels) as a within‐subjects factor and group as a between‐subjects factor. Active lever responses during SA, relapse, and reinstatement were analyzed using similarly structured ANOVAs. The propensity of the rats to escalate cocaine intake was measured by the escalation ratio, calculated as the ratio of drug infusions received on each day relative to the number of infusions received on the first extended access session, which provided a metric of the daily increase in cocaine intake.

In a similar manner to previously described,^20^, ^28^ instrumental performance in response to CS, shock, or drug presentation decreased throughout each 30‐minute block, such that animals had extinguished responding toward the end of each block. Thus, in order to assess the ability of cues, stress, or drug to reinvigorate extinguished responding more accurately, the first 10 minutes of each reinstatement block were compared with the last 10 minutes of the preceding block. Where applicable data were subject to an arcsine transformation to limit the impact of an artificial ceiling (ie, 100%). For all analyses, upon confirmation of main effects, differences among individual means were analyzed using Newman‐Keuls post hoc test.

Between‐subjects comparisons were further supported by dimensional analyses using Person *r* correlations. The escalation ratio used in between‐subjects analyses and dimensional analyses was that of the last SA session. The propensity of animals to relapse to cocaine‐seeking responding was measured as the total number of active lever responses during the 90‐minute extinction period.

For all analyses, significance was accepted at *α* ≤ 0.05, analyses for which *α* ≤ 0.1 was described as trends. Effect sizes are reported using partial *η* ^2^ (pη^2^).^29^

## 3 RESULTS

One animal died during surgery, and a problem with computer recording meant the data from one animal were lost for the second rGT. As the difference between the first and second rGT was critical for our grouping criteria, data from this animal were excluded.

**Figure 1:**
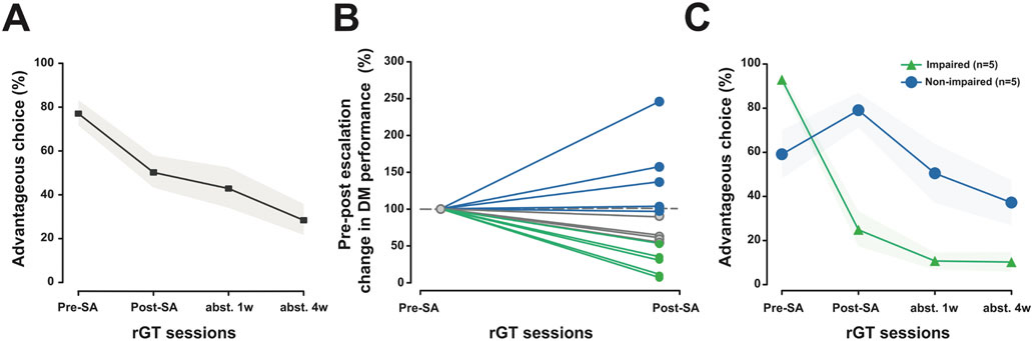
Individual variability in cocaine escalation‐induced change in decision making (DM). A, Escalation of cocaine self‐administration (SA) resulted in alterations to cost‐benefit DM at the population level that persisted throughout abstinence. B, Rats demonstrated pronounced interindividual differences in DM following escalation of cocaine intake. The effects of cocaine on DM were compared for each rat to a normalized score from the first session. The majority of animals showed impaired performance with a subset displaying unaffected or even improved performance. C, Rats were separated into terciles based on the alteration in advantageous choices following cocaine exposure and classified as displaying either improved/unaffected or impaired performance in DM. The impaired group demonstrated substantial decreases in advantageous choice following cocaine exposure that was not remediated after 1 month of abstinence. In contrast, animals in the unaffected group displayed unaltered or even improved performance following escalation of cocaine intake. Subsequently after either a week or a month of abstinence, during test sessions 3 and 4, respectively, choice of the advantageous options decreased for both groups, although it still remained higher in the nonimpaired group. Data are presented as mean ± standard error of the mean (SEM) or individual data points. rGT, rat Gambling Task

**Figure 2:**
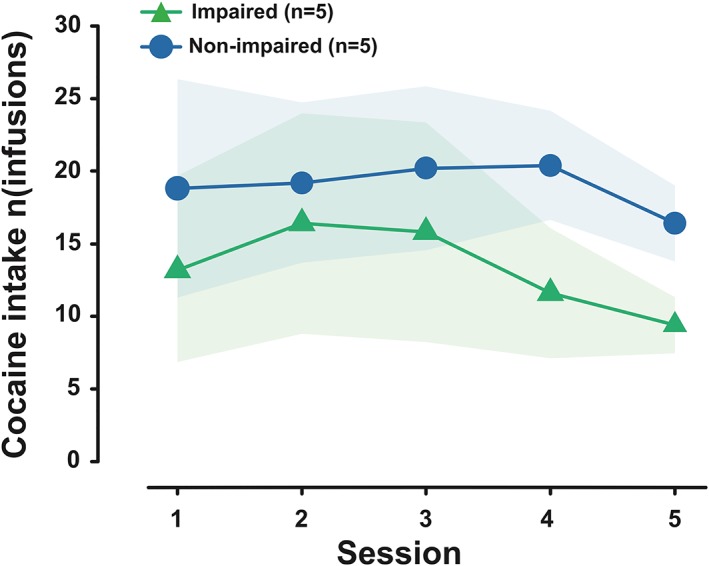
Impaired and unaffected rats displayed no differences in their acquisition of cocaine self‐administration over five short‐access sessions. Data are presented as mean ± standard error of the mean (SEM)

**Figure 3:**
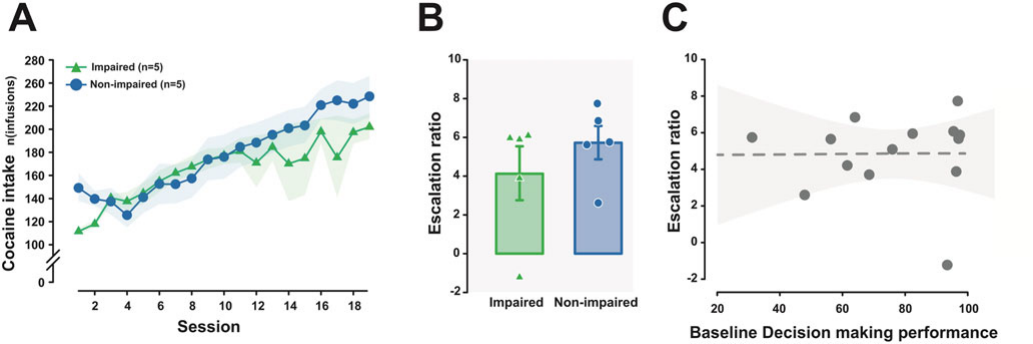
Individual differences in cocaine‐induced impairment in decision making are not associated with an increase in escalation of cocaine self‐administration. A, Rats in both the impaired and unaffected groups displayed a robust increase in the amount of cocaine self‐administered over the 19 daily 12‐h long‐access sessions, indicative of a loss of control over drug intake. B, Similarly, there was no difference in the escalation ratio on the last day of extended access between groups over extended access sessions, suggesting that there was no difference in the propensity to lose control over cocaine intake between the two populations. C, Escalation of cocaine self‐administration was not predicted by baseline decision‐making performance measured prior to drug exposure (the shaded area represents the 95% confidence interval). Data are presented as mean ± standard error of the mean (SEM) or individual data points

The rGT requires animals to assimilate information about the four available “decks” across the course of a single session. In order to maximize reward, animals must learn to avoid the high‐reward “decks” as these are associated with longer time‐out punishments and rather select from the “decks” offering lower immediate rewards but less severe time‐out punishments. During the initial session, the majority of animals learnt this strategy and eventually selected from the advantageous “decks” 76% of the time, with poor and good decision makers, in the lower and upper tercile of the population, making 52% ± 5.9 and 96% ± 0.34 advantageous choices, respectively. In order to determine the effects of escalated cocaine intake on decision making, rats were tested again on the rGT 1 day, 1 week, and 1 month after 19 sessions of extended access to cocaine SA. Cocaine exposure broadly impaired animals' decision making on the rGT, with advantageous choice decreasing across the four sessions (Figure 1A) (main effect of session: *F*_3,42_ = 8.93, *P* = 0.0002, pη^2^ = 0.39). However, there were pronounced individual differences in the degree to which cocaine exposure altered decision making on the rGT (Figure 1B). Thus, rats were stratified according to the change in decision‐making score from rGT1 to rGT2, as this reflects the impact of cocaine exposure on decision making since they were tested only 1 day after cessation of SA. As we were principally interested in investigating the effects of interindividual differences in cocaine escalation on subsequent decision making, animals were split into terciles and the upper and lower terciles as nonimpaired and impaired, respectively. The final number of animals included in subsequent between‐group comparisons was five that showed impaired and five that showed improved or unaffected decision making following cocaine SA, or five good and five poor decision makers stratified prior to drug exposure. Significant differences in advantageous choice were observed between the impaired and unaffected groups (main effect of session: *F*_1,8_ = 31.89, *P* < 0.0001, pη^2^ = 0.80; and session × group interaction: *F*_3,24_ = 20.15, *P* < 0.0001, pη^2^ = 0.72). Post hoc analyses revealed that there were significant differences between the two groups on three of the four rGT sessions (rGT 1: 0.90, *P* = 0.03; rGT 2: 1.09, *P* = 0.008; rGT 3: 0.79, *P* = 0.03; rGT 4: 0.64, *P* = 0.09) (Figure 1C). Impaired and unaffected rats displayed no differences in their acquisition of cocaine SA over five short‐access sessions (Figure 2) (main effect of group: *F*_1,8_ = 1.0, *P* = 0.35, pη^2^ = 0.11; and group × session interaction: *F*_4,32_ = 0.14, *P* = 0.97, pη^2^ = 0.02). Likewise, both groups exhibited a robust escalation in cocaine intake across 19 daily extended access sessions (Figure 3A) (main effect of group: *F*_1,8_ = 0.49, *P* = 0.51, pη^2^ = 0.06; session: *F*_18,144_ = 14.33, *P* < 0.0001; and group × session interaction: *F*_18,144_ = 1.48, *P* = 0.11, pη^2^ = 0.16). There was also no difference in the escalation ratio between the groups (Figure 3B) (main effect of group: *F*_1,8_ = 0.95, *P* = 0.36, pη^2^ = 0.11). Thus, impaired and unaffected rats did not differ in their propensity to acquire or escalate cocaine SA. The magnitude of the escalation of cocaine intake over time did not differ between rats stratified as good or poor decision makers prior to drug exposure (*F*_1,8_ = 0.777, *P* = 0.404, pη^2^ = 0.088) (data not shown) nor was it predicted by baseline decision‐making performance across the entire population (Figure 3C) (*R* = 0.01, *P* = 0.98). Therefore, preexisting individual differences in decision making did not contribute to individual propensity to escalate cocaine SA.

**Figure 4:**
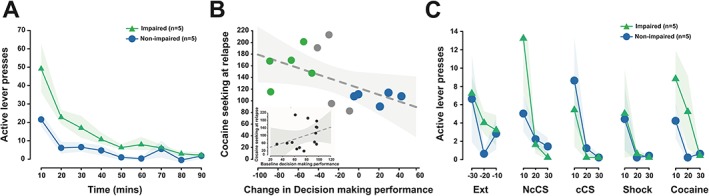
Cocaine‐induced decision‐making impairment predicts increased propensity to relapse but no differential sensitivity to cue‐, stress‐, or drug‐induced reinstatement. A, Both groups of rats showed an increase in responses on the lever previously associated with cocaine when they were reintroduced to a cocaine‐associated context. However, rats that were susceptible to the deleterious effects of cocaine on decision making demonstrated a higher propensity to relapse to drug seeking following a week of abstinence. B, Critically, the propensity of animals to persist in drug seeking during the relapse challenge under extinction conditions was predicted by the impairment in decision making performance following cocaine exposure but not baseline performance in decision making measured prior to drug exposure (insert) (shaded areas represent the 95% confidence interval). C, However, decision‐making impaired rats did not differ from nonimpaired rats in their propensity to reinstate extinguished responding after conditioned stimuli (CS), shock, or cocaine exposure. Data are presented as mean ± standard error of the mean (SEM) or individual data points

However, rats with impaired decision making were more vulnerable to relapse following forced abstinence than unaffected rats. Impaired rats displayed higher levels of instrumental responding over the course of a 90‐minute relapse challenge session under extinction carried out after 7 days of forced abstinence (Figure 4A) (main effect of time: *F*_8,64_ = 13.35, *P* < 0.0001, pη^2^ = 0.63; group: *F*_1,8_ = 13.43, *P* = 0.006, pη^2^ = 0.63; and group × time interaction: *F*_8,64_ = 2.24, *P* = 0.04, pη^2^ = 0.22). Post hoc tests revealed that this augmented response was only significant during the first 10‐minute time bin (time bin 1: 50.0, *P* = 0.0002, all other time bins: NS), likely reflecting that impaired animals did not display an impaired ability to alter their behavior in response to new contingencies. Critically, this higher vulnerability to relapse observed in impaired rats was not predicted by the propensity to escalate cocaine intake at the population level (*R* = 0.065, *P* = 0.82). However, a marked correlation was found between the change in decision‐making score from the first to the second rGT and the level of responding on the active lever during the relapse challenge for the entire population of 14 rats (Figure 4B) (*R* = −0.53, *P* = 0.05). The relationship between active lever presses at relapse was specific to the change in rGT score, as poor and good decision makers stratified prior to drug exposure did not differ from each other in their performance at relapse (*F*_1,8_ = 1.28, *P* = 0.29, pη^2^ = 0.137) (data not shown) nor was there any relationship between responding during relapse under extinction conditions and the decision‐making score at baseline across the entire population (*R* = 0.35, *P* = 0.22) (Figure 4B insert).

Lastly, the propensity of rats that displayed impaired decision making to respond more on the active lever during a relapse test was not observed in the subsequent CS‐, stress‐, or drug‐induced reinstatement tests (Figure 4C). All rats increased active lever presses in response to both noncontingently (main effect of block: *F*_1,8_ = 4.06, *P* = 0.08, pη^2^ = 0.34) and contingently presented CS (main effect of block: *F*_1,8_ = 3.49, *P* = 0.1, pη^2^ = 0.30), albeit only at a trend level (Figure 4C). In contrast, footshock‐induced stress failed to alter behavior (main effect of block: *F*_1,8_ = 2.31, *P* = 0.17, pη^2^ = 0.22), but a significant increase in active lever responses was observed following a noncontingent experimenter administering single infusion of cocaine (Figure 4C) (main effect of block: *F*_1,8_ = 12.11, *P* = 0.008, pη^2^ = 0.60). Impaired and unaffected rats displayed no differences in these reinstatement challenges (main effects of group: all *F*'s > 2.2, NS).

## 4 DISCUSSION

Despite a rather small sample size, the results of this study, supported by large to very large effect sizes, further demonstrate that baseline cost‐benefit decision‐making performance in rats, like humans,^14^, ^15^, ^30^ shows large interindividual variability (present study, Daniel et al 23 and Rivalan et al 24). Critically, these data suggest that baseline decision making does not predict the propensity to acquire cocaine SA or the vulnerability to escalate cocaine intake. Instead, the magnitude of the deficits in decision making precipitated by escalated cocaine intake predicted subsequent propensity to relapse following forced abstinence. The observed results are in agreement with data from studies using human subjects, whose impairments in executive functioning after exposure to drugs were associated with increased vulnerability toward relapse following abstinence.^10^, ^11^ The present data are also congruent with the finding that in human subjects with substantial exposure to cocaine, the failure to acquire the optimal strategy on the IGT is associated with dropout from treatment.^12^

The present study expands this body of evidence by demonstrating that extended access to cocaine, and associated escalation of intake, led to heterogeneous deficits in subsequent decision making, with some rats displaying a massive decrease in performance and others showing unaffected or even improved performance during the second rGT session.

The observation that drug‐induced deficits in decision making confer an increased propensity to subsequently respond during a relapse challenge under extinction conditions is congruent with the finding that disadvantageous decision making following exposure to cocaine, on another iteration of the rGT, was associated with an increased propensity to acquire instrumental responding for a cocaine‐paired cue, acting as a conditioned reinforcer.^18^ However, this previous study suggested that rats that exhibited riskier patterns of decision making at baseline were more sensitive to the deleterious effects of cocaine on decision making.^18^ These data are in contrast to those of this study, which indicated that rather than preexisting differences, it is the extent to which animals are sensitive to the deleterious effects of cocaine on decision making that confer vulnerability to relapse following a week of forced abstinence. The contradictory findings between these two versions of the rGT may be attributable to differences in training and testing between the two paradigms. Here, we used a version of the rGT wherein animals are required to learn about the relative utility of the various options across the course of a single session, consistent with the human IGT.^14^ This single‐session approach captures key elements of cost‐benefit decision making but does not enable the measurement of stable performance. Without this stable baseline, which controls for robust changes in decision making between rGT sessions, performance under the present conditions may potentially be more malleable. Consequently, the profound alterations in performance observed immediately following escalation of cocaine intake could be due to underlying differences in the strategies deployed by individual rats to acquire the task. It could therefore be argued that the putatively drug‐induced alterations observed here may be reflective of animals failing to adequately learn the contingencies and rather be attributable to a regression toward the mean. Contrary to such a suggestion, the impaired group actually performed better on the initial rGT demonstrating that they readily acquired an optimal strategy prior to drug exposure. Additionally, we and others have repeatedly shown that rats can adjust their strategy in response to alterations in contingencies across multiple test sessions.^23^, ^27^, ^31^ Lastly, both groups showed broadly consistent choice throughout the remaining sessions, which is at odds with the more pronounced shifts that would be expected if performance was stochastic and reflected a regression toward the mean.

Although it should be noted that despite broadly consistent performance, all rats performed incrementally worse over time, a finding that parallels the long‐lasting cognitive deficits that cocaine appears to induce in human (see Rogers and Robbins 32 for review). Long‐lasting deficits have also been reported in other animal studies,^33^ although these findings are not unequivocal as a recent study showed that stimulant‐induced deficits were remediated over a time course similar to the one used here.^34^ These apparent discrepancies are likely related to differences in inter‐testing training, which can have a pronounced effect on the cognitive process taxed (see Cocker and Winstanley 35 for discussion). Nevertheless, the finding that all animals continue to get worse following the cessation of cocaine SA is not attributable to the acute psychoactive effects of the drug but may reflect the long‐term consequences of a history of escalated cocaine intake. One potential explanation may be an increase in risk tolerance, as animals are repeatedly exposed to testing. Indeed, exposing animals to unpredictable schedules of reward increases risky decision making.^36^ However, animals have previously been shown to demonstrate consistent performance across multiple test days on the rGT, indicating that increased risk tolerance alone is insufficient to account for the increase in disadvantageous choice and rather exposure to cocaine appears to be critical in instantiating or exacerbating these negative effects.

The increased propensity to respond during the relapse challenge under extinction conditions displayed by the impaired group is potentially suggestive of the cognitive processes that underlie the alterations in decision making. One potential process contributing to both deficits in cost‐benefit decision making and higher propensity to relapse may be impulsivity. Indeed, continued responding during extinction has been suggested to arise as a result of disinhibition; ie, rats are unable to withhold a prepotent motor response toward a stimulus (here, the active lever) previously associated with a reinforcer.^37^ Increased impulsivity has also been associated with poor cost‐benefit decision making on a rodent version of the IGT.^38^ However, there were no differences in decision making or the escalation of cocaine SA between impaired and nonimpaired rats. Consequently, as high impulsivity trait has been shown to exacerbate the escalation of cocaine intake,^39^, ^40^, ^41^ *de novo* differences in impulsivity are unlikely to have contributed to the increased propensity to relapse observed in impaired rats.

A cocaine‐induced impairment in cognitive flexibility leading to increased preservative responding could also have potentially contributed to the deficits in decision making and the higher propensity to relapse displayed by impaired rats. Indeed, cocaine exposure has been suggested to result in inflexible decision making due to impairments in updating associative information.^42^ Relatedly, a recent study has shown that chronic exposure to the stimulant methamphetamine impaired flexible decision making, with rats continuing to select previously advantageous options, due to a deficit in using negative outcomes to effectively guide behavior on a reversal learning task.^34^ These data indicate that impaired rats in the present study are unable to switch their behavior away from the options that were initially advantageous. This may arise from animals assigning increased motivational valence to the larger rewards or a decrease in the potency with which the aversive properties of time‐out punishments facilitate switching between options. Interestingly, recent data have suggested that even in the absence of drug, a small subset of animals can become relatively inflexible following the first test day on the rGT. This pattern of behavior was correlated with a decreased sensitivity to contingency degradation, suggesting that these rats were more predisposed toward the development of habitual behaviors.^31^ Consistent with this observation, acute cocaine injections post training have been shown to facilitate habitual control over instrumental responding for natural reinfocers.^43^ This raises the possibility that cocaine SA here may have facilitated the formation of rigid habit‐based strategies in the rGT in a subset of animals. Whether the deleterious effects of cocaine on decision making in the impaired group and the augmented instrumental response during extinction in the relapse challenge are due to an increase in impulsivity or preservative responding linked to an increase in habit formation is unclear, even if habits are not necessarily more resistant to extinction than goal‐directed behaviors. Further investigations aiming to elucidate the psychological and neural basis of these behavioral manifestations may be beneficial in guiding future treatment strategies.

Recent data from our lab suggest that the behavioral deficits displayed by impaired rats may depend on drug‐induced alterations of the anterior insular cortex (AIC). Thus, the AIC supports the acquisition of optimal exploitation strategies in the rGT and contributes to high impulsivity trait and the associated increased propensity to develop compulsive behaviors.^23^, ^44^ Lastly, we recently demonstrated that the AIC bidirectionally controls the escalation of cocaine SA.^20^ Taken together, these results could imply that individual differences in drug‐induced impairment of AIC function may confer vulnerability toward drug‐induced deficits in cost‐benefit decision making and associated increased propensity to relapse.^45^

Overall, the present study demonstrates that escalated cocaine SA greatly influences subsequent individual ability to optimize reward in a cost‐benefit decision‐making task. Despite marked interindividual differences in subsequent performance, individuals that showed the worst impairments were more likely subsequently to relapse after a period of abstinence. Moreover, the degree to which cocaine deleteriously impacted decision making predicted subsequent vulnerability to relapse. Ultimately, these data suggest that the canonical decision‐making deficits observed in human drug addiction are not a preexisting trait but rather arise as a result of the neurobiological sequela of chronic drug use and contribute to the subsequent chronicity of the disorder.

## AUTHORS CONTRIBUTION

J.Y.R., M.L.D., A.B.R., and D.B. designed the experiment. J.Y.R. and M.L.D. carried out the experiments. J.Y.R., A.B.R., P.C., and D.B. analyzed the data. P.C. and D.B. wrote the paper. A.B.R. and J.Y.R. offered intellectual input to the final manuscript.
